# Clinical effect of glucosamine hydrochloride combined with compound osteopeptide injection for knee osteoarthritis

**DOI:** 10.12669/pjms.39.6.8151

**Published:** 2023

**Authors:** Mo Han Liu, Kang He, Fengxia Liu

**Affiliations:** 1 Mo Han Liu, Department of Allergy, Shandong Weifang People’s Hospital, Weifang 261041, Shandong Province, P.R. China; 2 Kang He, Department of Allergy, Shandong Weifang People’s Hospital, Weifang 261041, Shandong Province, P.R. China; 3 Fengxia Liu, Department of Allergy, Shandong Weifang People’s Hospital, Weifang 261041, Shandong Province, P.R. China

**Keywords:** Compound osteopeptide injection, Glucosamine hydrochloride, Knee osteoarthritis

## Abstract

**Objective:**

To investigate the clinical efficacy of glucosamine hydrochloride combined with compound osteopeptide injection for knee osteoarthritis (KOA).

**Methods:**

We retrospectively collected clinical data of 82 patients with KOA admitted to Shandong Weifang People’s Hospital from April 2019 to September 2022. According to the treatment records, 35 patients received an intramuscular injection of compound osteopeptide (control group), and 47 patients received an injection of glucosamine hydrochloride combined with compound osteopeptide (observation group). We compared clinical efficacy, WOMAC scores, inflammatory factor and CD4^+^ and CD8^+^ levels, and the incidence of adverse reactions between the two groups.

**Results:**

The observation group’s total efficacy (95.74%) was significantly higher than the control group’s (80.00%; *P*<0.05). Treatment led to a significant reduction in WOMAC scores in both groups. In addition, the levels of tumor necrosis factor (TNF-α) and interleukin-6 (IL-6) in the observation group were significantly lower than those in the control group (*P*<0.05); while the levels of CD4^+^ and CD8^+^ were significantly higher in the observation group (*P*<0.05).

**Conclusions:**

Compared with compound osteopeptide injection alone, glucosamine hydrochloride combined with compound osteopeptide injection is more effective for patients with KOA, with improved level of inflammatory factors and immune function.

## INTRODUCTION

Knee osteoarthritis (KOA) is a degenerative disease with articular cartilage degeneration and bone hyperplasia with global prevalence of 16%.^1^,^2^ The clinical symptoms include mainly swelling and knee pain, but the disease can progress to include knee joint deformity and dysfunction.^3^ Clinical research has mostly focused on articular cartilage degeneration treatments. Interventions to control inflammation and nourish the cartilage can relieve pain and delay the progress of the disease.^4^,^5^

Patients with early or middle stage KOA usually receive conservative treatment. Compound osteopeptide injections are commonly used, they contain bone polypeptides, amino acids, cytokines, and other components, which can stimulate bone growth and regulate bone metabolism.^6^ Studies have shown that improving the microcirculation of the affected area and protecting the nerves is also important during KOA treatments. Therefore, two or more drugs should be combined to improve the efficacy of the osteopeptide injections.^7^,^8^ Glucosamine is an essential component that delays the breakdown of cartilage and repairs damaged cartilage. Its protective effects on articular cartilage and promotion of knee joint function have been studied.^9^ All above form the basis of combined KOA treatments.

The literature on the influence of glucosamine hydrochloride and compound bone peptide injection on the levels of inflammatory factors and immune function-related indicators in patients with KOA is limited. Therefore, we designed this study to investigate the effects of the combined therapy on these indicators.

## METHODS

We retrospectively collected data from 82 patients with KOA treated in Shandong Weifang People’s Hospital from April 2019 to September 2022, including 32 men and 50 women. The average age of the participants was 55.90±6.40 years, the mean BMI was 22.40±2.45 kg/m^2^, and the mean course of disease was 16.56±7.01 months. The records showed that 35 patients received intramuscular injections of compound osteopeptide (control group); while 47 patients received glucosamine hydrochloride combined with compound osteopeptide injection (observation group).

### Inclusion criteria:

- Participants had diagnoses that met KOA diagnostic criteria.^10^
- All participants were aged from 18 to 76 years.
- All participants had a unilaterally affected knee joint.
- Participants with Kellgren-Lawrence grade 1, 2 or 3.
- All participants had complete medical records.

### Exclusion criteria:

- Patients with rheumatoid arthritis or tuberculosis of knee joints.
- Patients with major organ dysfunction or severe underlying diseases in heart, lung, liver, and kidney.
- Patients with malignant tumors.
- Patients with active peptic ulcers.
- Patients who have previously received glucosamine hydrochloride or compound osteopeptide injection.
- Patients with contradiction to glucosamine hydrochloride or / combined with compound osteopeptide injection
- Pregnant or lactating women.

### Ethical Approval:

The medical ethics committee of our hospital approved the study (Approval number, 2023XX-029; date, 2023-02-22).

Patients in the observation group received compound osteopeptide (Changzhou Fangyuan Pharmaceutical Co., Ltd, H20054006, Specification: 5ml:75mg) 4-mL intramuscular injections, once a day for four weeks; and oral glucosamine hydrochloride tablets (Jiangsu Zhengda Qingjiang Pharmaceutical Co., Ltd, H20060647, Specification: 0.75g*90 tablets) 0.75 g three times/day for four weeks. Patients in the control group received only the compound osteopeptide injections during the same period. In addition, all patients received non-drug treatments such as physical therapy, knee joint functional training, and joint health knowledge education. We extracted basic clinical data and relevant clinical indicators of patients from their records including.

### Treatment outcome data:

Recovery (disappearance of symptoms such as joint pain, swelling, and stiffness, joint function, and deformity corrections), effective treatment (alleviated swelling, stiffness, and joint rattle, improved joint function, and deformity correction), or lack of effect (lack of improvement of symptoms or joint function, sustained deformity). In addition, we calculated the total efficacy = (number of patients with recovery + number of patients with effective treatment)/Total number of cases.

### Osteoarthritis index scores:

Western Ontario and McMaster Universities Osteoarthritis Index (WOMAC) was used to evaluate the condition and functional status of patients with KOA.^11^ The scale includes three dimensions (24 items): Pain (five items); stiffness (two items), and joint function (17 items). Each item is scored with a five-level scoring method (none, slight, medium, serious, and very serious), with a score of 0–4 points, and the total score of the WOMAC scale is 0–96 points. The higher the score, the more serious the disease.^12^

### Levels of tumor necrosis factor:

Tumor necrosis factor alpha (TNF-α), interleukin-6 (IL-6), CD4^+^ and CD8^+^ before and after treatment were measured from supernatants of 5-ml of venous blood samples centrifuged at 3500 r/minute for 15 minutes. The levels of TNF-α and IL-6 were detected using enzyme-linked immunosorbent assays (kit provided by Shanghai Huzheng Biotechnology). The levels of CD4^+^ and CD8^+^ were detected by flow cytometry (Eckman-Coulter Epics XL).

### Adverse reactions:

After the medication, it included constipation, low fever, rash, and any other signs or symptoms were observed.

### Statistical analysis:

We processed the data with SPSS22.0 to conduct a statistical analysis. Non-grade count data were expressed as percentages [n (%)], and compared using *χ^2^* inspection. The measurement data with normal distribution were expressed as means (*χ̅*±*S*), and compared using *t*-tests. We considered *P-values* <0.05 as statistically significant.

## RESULTS

We analyzed data from 82 patients: 35 in the control group, 16 men and 19 women, with a mean age of 55.34±6.38 years, mean BMI of 24.12±2.35 kg/m^2^, mean course of disease of 16.14±7.29 months, 18 left knees affected, and 17 right knees affected; and, 47 patients in the observation group, 18 men and 19 women, with a mean age of 56.32±6.44 years, mean BMI of 24.61±2.52 kg/m^2^, mean course of disease of 16.87±6.85 months, 27 left knees affected, and 20 right knees affected. We found similar baseline clinical data between the patients of the two groups (*P*>0.05; Table-I). The total clinical efficacy of the observation group at 95.74% was significantly higher than the 80.00% rate of the control group (*P*<0.05; Table-II). Before treatment, the WOMAC scores and total scores of the two groups were similar (*P*>0.05). After treatment, the WOMAC scores and total scores reduced in both groups, but the observation group had significantly greater reduction (*P*<0.05; Table-III). Before treatment, the levels of TNF-α, IL-6, CD4^+^ and CD8^+^ were similar between the patients of the two groups (*P*>0.05). After treatment, the levels of TNF-α and IL-6 decreased in both groups, but the mean levels were significantly lower in the observation group (*P*<0.05).

**Table-I:** Comparison of basic clinical data between two groups.

Group	n	Gender (Male/Female)	Age (years)	BMI (kg/m²)	Course of disease (months)	Affected side (n)

						Left/Right
Control	35	14/21	55.34±6.38	24.12±2.35	16.14±7.29	18/17
Observation	47	18/29	56.32±6.44	24.61±2.52	16.87±6.85	27/20
*χ^2^*/*t*	-	0.024	-0.681	-0.892	-0.464	0.293
*P*	-	0.876	0.498	0.375	0.644	0.588

**Table-II:** Comparison of clinical effects between the two groups [n (%)].

Group	n	Recovery	Effective	No effective	Total efficiency (%)
Control	35	10	18	7	80
Observation	47	22	23	2	95.7
*χ^2^*	-	-	-	-	6.266
*P*	-	-	-	-	0.044

**Table-III:** Comparison of WOMAC scale scores between the two groups before and after treatment (*χ̅*±*S*).

Group (*n*)	Pain score		Stiffness score		Joint function score		Total score	

	Before treatment	After treatment	Before treatment	After treatment	Before treatment	After treatment	Before treatment	After treatment
Control (*n*=35)	7.82±2.13	4.94±1.75*	3.17±1.07	1.97±0.57*	28.03±3.08	18.74±2.60*	39.03±3.69	25.66±3.19*
Observation (n=47)	8.02±2.10	3.96±1.78*	3.36±1.13	1.28±0.65*	29.15±3.22	15.62±2.50*	40.53±4.10	20.85±3.33*
*t*	-0.408	2.498	-0.771	5.054	-1.586	5.503	-1.714	6.581
*P*	0.684	0.015	0.443	<0.001	0.117	<0.001	0.090	<0.001

***Note:***

* P < 0.05 *vs*. same group before treatment.

The levels of CD4^+^ after treatment were higher than before treatment in both groups, and the observation group had the highest mean level (*P*<0.05; Table-IV). After treatment, we found a report for one case of low fever and one case of rash in the control group with incidence of adverse reactions of 5.7%. The observation group had one case of constipation, one case of low fever, and one case of rash for an incidence of adverse reactions of 6.4%. No statistically significant difference was found between the groups (*P*>0.05).

**Table-IV:** Comparison of inflammatory factor, CD4^+^ and CD8^+^ levels between the two groups (*χ̅*±*S*)

Group (*n*)	TNF-α (ng/L)		IL-6		CD4^+^(%)		CD8^+^(%)	

	Before treatment	After treatment	Before treatment	After treatment	Before treatment	After treatment	Before treatment	After treatment
Control (*n*=35)	88.43±6.64	46.23±4.94*	85.31±5.99	44.03±4.88*	33.03±2.92	36.37±3.40*	18.97±2.72	16.88±2.81*
Observation (*n*=47)	87.49±7.18	34.15±4.81*	86.70±6.22	34.57±5.99*	32.30±2.63	41.70±3.48*	18.44±3.06	13.47±3.30*
*t*	0.605	11.113	-1.015	7.635	1.188	-6.921	0.806	4.936
*P*	0.547	<0.001	0.313	<0.001	0.238	<0.001	0.423	<0.001

***Note:***

* P < 0.05 *vs*. same group before treatment.

## DISCUSSION

Our results showed that glucosamine hydrochloride combined with compound osteopeptide injection had better therapeutic effects in patients with KOA than the injection of compound osteopeptide alone. The total WOMAC scale scores in the observation group were significantly lower than those in the control group, a finding consistent with those by Hu T et al.^13^ Research on the causes of KOA suggests that osteophytes in the knee joint cavity induce local inflammation and cause swelling and pain, limited movement, and other symptoms.^14^,^15^

KOA is an inflammatory disease with many inflammatory factors expressed at a high levels. TNF-α is a pro-inflammatory factor that participates in local tissue inflammatory reactions.^16^ Li J et al^17^ showed that the TNF-α levels are increased in patients with KOA in direct proportion to the severity of the disease. TNF-α can change the living environment of chondrocytes, promote the proliferation of synovial fibroblasts, promote formation of fibroid lesions in synovial tissue and soft bone lesions, lead to matrix degradation, cause the destruction of articular cartilage, and accelerate its degeneration. IL-6 is another inflammatory factor shown to be highly expressed in patients with KOA initially during the early stages of the disease and becoming more abundant with the disease progression.^18^,^19^ Chen Y et al.^20^ showed that the levels of IL-6 are significantly higher in patients with KOA than in healthy individuals. IL-6 can promote the proliferation of synovial cells, the production of matrix metalloproteinases in synovium, and the destruction of cartilage.

In addition, Groves-Williams D et al.^21^ showed that infiltrating T lymphocytes in patients with KOA do not play a role in immune regulation and instead are abnormally activated and proliferate, resulting in the release of a large number of inflammatory factors that cause the destruction of the cartilage matrix. In our study, we measured the levels of TNF-α, IL-6, and CD4^+^ and CD8^+^ lymphocytes as markers of disease. We found that the total efficacy of patients receiving glucosamine hydrochloride combined with compound osteopeptide injection was significantly higher than that in the control group.

Moreover, the TNF-α and IL-6 levels were lower, and the counts of CD4^+^ and CD8^+^ were higher in the observation group than in the control group. These results are similar to those in the study by Wang S et al,^22^ and they suggest that the treatment of KOA with glucosamine hydrochloride combined with compound osteopeptide injection improves the treatment efficacy, reducing the inflammation. The study of Baria MR et al.^23^ showed that when glucosamine hydrochloride combined with compound osteopeptide injection is used to treat patients with KOA, it can effectively reduce the expression level of inflammatory factors, inhibiting inflammation and oxygen free radical damage, reducing the expression level of MMPs, inhibiting the cartilage damage, improving the immune function, and increasing the levels of CD4^+^ and CD8^+^, a finding consistent with ours. In addition, we also found few adverse drug reactions and no serious adverse events during the treatment (without significant differences between the study groups) indicating that the treatment scheme of glucosamine hydrochloride combined with compound osteopeptide injection is relatively safe.

The study of Liu J et al.^24^ showed that the treatment with glucosamine hydrochloride combined with compound osteopeptide injection was ideal for patients with KOA without increases in adverse reactions due to the combination of the drugs. However, most patients with KOA are middle-aged and elderly individuals with underlying diseases, who may be more sensitive to adverse drug reactions than others. Therefore, we suggest that patients need to be comprehensively evaluated considering their physical condition and drug hypersensitivity history before initiating the combined regimen for KOA, and that education and guidance on safe drug use, and follow-ups and monitoring of adverse reactions during the treatment are needed to achieve a smooth implementation of this treatment plan, which seems to provide a clear clinical benefit for patients with KOA.^22^,^24^

### Limitations of the study:

This was a single-center retrospective study with a small population and inevitable selection bias. We focused on few serological indicators and are aware of the possibility of information bias. Our findings are mostly restricted to specific cases and one-sided. Moreover, the adverse events and recurrence rate of pain should be further investigated in future studies.

## CONCLUSION

Compared with compound osteopeptide injection alone, glucosamine hydrochloride combined with compound osteopeptide injection is more effective for patients with KOA, with improved level of inflammatory factors and immune function.

### Authors’ contributions:

**ML:** Conceived and designed the study.

**KH** and **FL:** Collected the data and performed the analysis.

**ML:** Was involved in the writing of the manuscript and is responsible for the integrity of the study.

All authors have read and approved the final manuscript.
